# Interdisciplinary Methods for Zoonotic Tissue Acellularization for Natural Heart Valve Substitute of Biomimetic Materials

**DOI:** 10.3390/ma15072594

**Published:** 2022-04-01

**Authors:** Roman Major, Magdalena Kopernik, Roman Ostrowski, Piotr Wilczek, Amanda Bartkowiak, Karolina Szawiraacz, Grzegorz Lis, Janusz Lekki, Maciej Gawlikowski, Łukasz Major

**Affiliations:** 1 Institute of Metallurgy and Materials Science, Polish Academy of Sciences, 25 Reymonta st., 30-059 Cracow, Poland; r.major@imim.pl (R.M.); k.szawiraacz@imim.pl (K.S.); l.major@imim.pl (Ł.M.); 2 AGH University of Science and Technology, Al. Mickiewicza 30, 30-059 Cracow, Poland; 3 Institute of Optoelectronics, Military University of Technology, Gen. S. Kaliskiego st. 2, 00-908 Warsaw, Poland; roman.ostrowski@wat.edu.pl; 4 The President Stanislaw Wojciechowski Calisia University, Nowy Swiat 4 st., 62-800 Kalisz, Poland; p.wilczek@frk.pl; 5 Institute of Nuclear Physics Polish Academy of Sciences, PL-31342 Krakow, Poland; amanda.bartkowiak@ifj.edu.pl (A.B.); janusz.lekki@ifj.edu.pl (J.L.); 6 Department of Histology, Jagiellonian University Medical College, Kopernika 7, 31-034 Krakow, Poland; mmlis@cyf-kr.edu.pl; 7 Department of Biosensors and Processing of Biomedical Signals, Faculty of Biomedical Engineering, Silesian University of Technology, Roosevelt Str. 40, 41-800 Zabrze, Poland; maciej.gawlikowski@polsl.pl

**Keywords:** aortic valve tissue, pericardium, hyperelastic material model, decellularized tissue scaffold, proton microbeam irradiation, tissue autofluorescence, elastic fibers, laser-tissue interaction

## Abstract

The goal of this work was to create a bioactive tissue-based scaffold using multi-disciplinary engineering materials and tissue engineering techniques. Materials & methods: Physical techniques such as direct laser interference lithography and proton radiation were selected as alternative methods of enzymatic and chemical decellularization to remove cells from a tissue without degradation of the extracellular matrix nor its protein structure. This study was an attempt to prepare a functional scaffold for cell culture from tissue of animal origin using new physical methods that have not been considered before. The work was carried out under full control of the histological and molecular analysis. Results & conclusions: The most important finding was that the physical methods used to obtain the decellularized tissue scaffold differed in the efficiency of cell removal from the tissue in favour of the laser method. Both the laser method and the proton method exhibited a destructive effect on tissue structure and the genetic material in cell nuclei. This effect was visible on histology images as blurred areas within the cell nucleus. The finite element 3D simulation of decellularization process of the three-layer tissue of animal origin sample reflected well the mechanical response of tissue described by hyperelastic material models and provided results comparable to the experimental ones.

## 1. Introduction

Tissue of animal origin defects remains one of the most serious problems in cardiac surgery. Defective valves can be replaced by biological (natural) or mechanical (artificial) implants, although both approaches have several drawbacks [1,2,3]. Generally, biological implants suffer from calcifications, which evoke inflammatory reactions and infections [4,5]. Instead, artificial mechanical valve implants are characterized by high durability but simultaneously they need continuous anticoagulant therapy [4]. Combining techniques of materials science and tissue engineering seems to be a promising tool to develop a new kind of material that could be finally used to create new generation of tissue of animal origin bioprosthesis. Current advanced tissue and organ transplantation is focused on the development of autologous tissue material. This goal can be achieved by tissue engineering methods utilizing xenogenic decellular biodegradable matrix populated by autologous cells. Biografts of this kind should be free from previously described complications typical for currently used prostheses. Moreover, materials dedicated for bioprostheses should exhibit potential for growth, self-repair, and remodeling similar to the native tissues.

New ways of tissue manufacturing rely on the development of an autologous tissue material, e.g., bioprosthesis obtained through materials science and tissue engineering techniques in which the artificial scaffold is composed of decellular (without cells) matrix with the patient’s own cells (autologous cells). Such implants would be free of complications occurring when current prostheses are used, such as calcification, inflammation reactions, increased susceptibility to infections, necessity for anticoagulant medicaments or low durability. Natural scaffolds consisting of decellularized biological tissue have been successfully used in the construction of the prosthetic valves [6]. In such case, calcification and structural changes in tissue are the most difficult issues occurring when the biological valve prosthesis is used [7]. Immune response to such implant is a limiting factor in the application of the prosthetic tissue of animal origin [8]. The need for long term anticoagulant therapy may, however, negatively affect the use of mechanical prostheses. New therapy involves seeding the decellularized tissue scaffold with patient’s autologous cells. The animal-origin prosthetic valve could have the potential for growth, self-repair, and reconstruction, similar to native human tissue. A complete removal of cells from tissue, while maintaining intact the native structure of extracellular matrix (ECM) is very important during preparation of decellularized valve leaflets [9]. Such natural scaffolds express immune-modulatory effects in the body, which may influence the host remodeling response [10,11,12,13,14,15].

There are several decellularization procedures that can be applied to remove cells and thereby reduce the amount of galactose-α-1,3-galactose (α-Gal) [16,17,18,19,20,21,22]. α-Gal is one of the constituents of mammalian cell membranes and is recognized by the immune system as a foreign body leading to organ rejection after transplantation. Therefore, the animal origin cardiac tissue, especially those derived from pigs that are free from α-Gal, can be an attractive source for tissue engineered animal origin prosthesis. Decellularization protocols may differ by the type of the elaborated tissue [23,24,25,26,27,28], the amount of the removed cells, or preservation level of extracellular matrix [29]. Unfortunately, using these protocols, it is impossible to remove all cells within the sample tissue. Prolonged times of incubation with the decellularizing agent can cause collagen disruption and changes in biomechanical properties. Effective decellularization methodology is dictated by the following factors: tissue density and organization, geometric and biological properties desired for the final product, and targeted clinical application. Tissue decellularization with good preservation of ECM integrity can be optimized by choosing the right decellularizing agents and protocols during the processing [30]. However, the development of new decellularization techniques and the advent of three-dimensional whole organ decellularization have since emerged. In addition, the deleterious in vivo effects of residual cellular material are becoming more recognized [31,32,33,34]. Currently, the most popular and extensively investigated protocols regarding decellularization processes include chemical and enzymatic methods [35,36,37,38,39,40,41,42,43,44,45,46,47,48,49,50]. However, there are lately emerging reports [51] indicating that these conventional methods used for decellularization are harsh in nature and destructive to essential components of the ECM structure.

The known physical methods applied in decellularization processes are: thermal shock, mechanical pressure, electroporation, perfusion, pressure gradient, superficial fluids, ultrasonic waves, immersion and agitation, and vacuum-assisted [51]. Yet, none of the current tissue engineering strategies have considered laser or proton irradiation as effective methods for decellularized scaffolds production [52]. Therefore, this study evaluates the usefulness of new physical methods to obtain animal-origin, cell-free, and intact ECM structure.

The aim of this work was not to eliminate conventional decellularization techniques but to find an alternative solution, which could be also introduced as a hybrid technique with the enzymatic one. Preliminary tests and basic analysis for the laser radiation effect on tissue structure were reported in our previous article [53].

The aim of the work was to create an appropriate substrate for the preparation of tissue-based scaffold populated by cells of graft recipient. The executed work consisted of a few stages aiming to create a fully functional tissue by multidisciplinary approach involving materials science, tissue engineering, and genetic engineering. Aortic valves of animal origin were utilized in the study and they will provide background to produce biocompatible tissue for transplantation into humans. Generally, foreign (host) cells are highly immunogenic components of a graft and are mostly responsible for its rejection. In order to remove cells from the biografts, aortic valves of animal origin were modified by two newly applied physical methods: direct laser interference lithography (DLIL) and proton radiation. Conventional chemical process was also applied to achieve decellularized tissue, which was used as a control sample. The degree of removed cells and degradation of ECM was controlled by histological and microscopic analysis. 

The numerical part of this work was dedicated to modelling of the decellularization process, which should help to analyze the effect of dynamic wave loading on the tissue of animal origin [54]. In the first approach [55], simulations were performed on single-layer samples with the use of rheological models identified by the authors. In the present paper, simulations were performed on three-layer samples with preservation of the histological structure of the tissue of animal origin using hyperelastic material models [56,57].

## 2. Materials and Methods

### 2.1. Tissue

Pig hearts were taken from full-grown animals weighing between 70 kg and 80 kg. The experimental hearts were placed in sterile containers in Ringer’s solution (Solutio Ringeri, FRESENIUS KABI, Bad Homburg, Germany) and transported on ice to the laboratory in less than 1 h. The aortic valve was removed from each heart. The samples were rinsed with Ringer’s solution to remove blood and incubated for 24 h at 4 °C in an antibiotic bath containing 1% penicillin/streptomycin (Gibco, Waltham, MA, USA), 0.1% Ciprinol (Krka, Warsaw, Poland), and 0.2% Mycomax (Zentiva, Prague, Czech Republic) dissolved in 1000 mL of Ringer’s solution.

### 2.2. Decellular Matrix Preparation

#### 2.2.1. Fe Model of Decellularization Process

**Figure 1:**
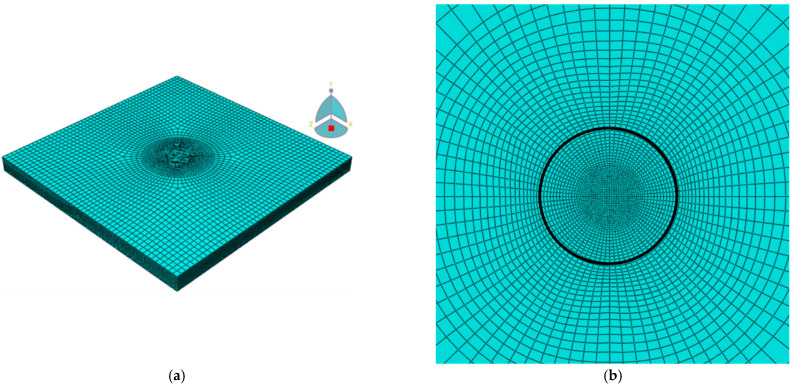
3D finite element mesh of three-layer tissue of animal origin model: (**a**) whole model—side view; (**b**) enlargement of central part of the model—top view.

The cross-section of the tissue of animal origin revealed the presence of three layers of tissue, i.e., fibrosa, spongiosa, and ventricularis. Applying the results of histological examinations and dimensions of samples, the following thicknesses were assumed for the individual layers: fibrosa—0.3 mm, spongiosa—0.2 mm, and ventricularis—0.16 mm. The accepted thicknesses for each layer of tissue were in ranges of values shown in the literature [58,59,60], where fibrosa had 46–58%, spongiosa had 23–30%, and ventricularis had 19–24% of the total thickness of the animal origin tissue. The tissue was modelled using cubic C3H8R finite elements (FEs), which are optimal for systems with simple or not too complicated geometry. Abaqus FE code generated meshes without mesh quality validation warnings and with very good mesh quality parameters that had 102,697 nodes and 95,040 elements. The FE mesh and the enlargement of the central part of the tissue model (place of applying load) is presented in Figure 1.

The simulations were performed using the Abaqus/Explicit solver to adjust the calculation performance to the dynamic impact of the tissue sample as well as to obtain more accurate results. The mesh nodes of the lower surface of the model were blocked in all displacement and rotation degrees of freedom (ENCASTRE boundary condition). The ENCASTRE boundary condition was adopted in order to obtain the stability of the solution. The maximum energy density (0.3 J cm^−3^) with a shockwave characteristic was applied as the load corresponding to the greatest energy density change, i.e., with a diameter of 1 mm on the upper surface of the sample. The simulation assumed that the sample was exposed to a load for 6 × 10^−7^ s, from the moment of its appearance until it completely subsides. The total duration of each simulation was set to 1.5 × 10^−4^ s.

The tissue density was taken as the density of 1.083 g cm^−3^ and was used for each layer of tissue. A Poisson’s number of 0.475 was assumed for each layer. Table 1 summarizes the elastic and hyperelastic parameters for the tissue materials [56,57] used in the simulations. As the table shows, the Ogden material model was used to model the hyperelasticity, in which the relationship between stress and strain results from the strain energy function *W*. For the Ogden material model, the strain energy function formula is as following:

(1) $$W=\overset{N}{\underset{i=1}{\sum }}\frac{\mu_{i}}{\alpha_{i}}\;\left(\lambda_{1}^{\alpha_{i}}+\lambda_{2}^{\alpha_{i}}+{\;\lambda }_{3}^{\alpha_{i}}-3\right)$$

where: *µ_i_*, *α_i_*—material constants, and *λ*_n_—components of deviatoric strain.

#### 2.2.2. Decellularization Using Chemical Methods

As a reference to the physical techniques studied within this work, the enzymatic method was applied as a conventionally used decellularization process. Tissues of animal origin were incubated at 37 °C for 24 h in a detergent solution of 250 mL in PBS. The detergent mixture contained: 0.25% Triton-X100 solution (X-100; Triton X-100; octylphenol ethylene oxide condensate; Octoxynol-9; toctylphenoxypolyethoxyethanol. Triton X-100 was a registered trademark formerly owned by Rohm and Haas Co., but now owned by Union Carbide); 0.25% sodium deoxycholate; and commercially available solutions: 2.5 mL × 1 mL Fluconazole, and 0.25 mL Ciprinol. After this process, samples were placed for 24 h at room temperature in PBS solution. Three successive washing steps with PBS were necessary to remove the detergent and cell residues. Each step of the decellularization procedure was prepared under shaking conditions. Tissue scaffolds were stored in an antibiotic bath at 4 °C and used within a week.

#### 2.2.3. Laser Method

##### Direct Laser Interference Lithography Experiments

**Figure 2:**
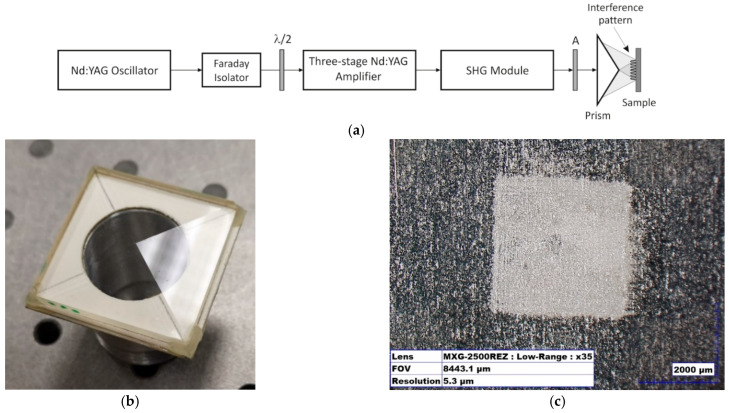

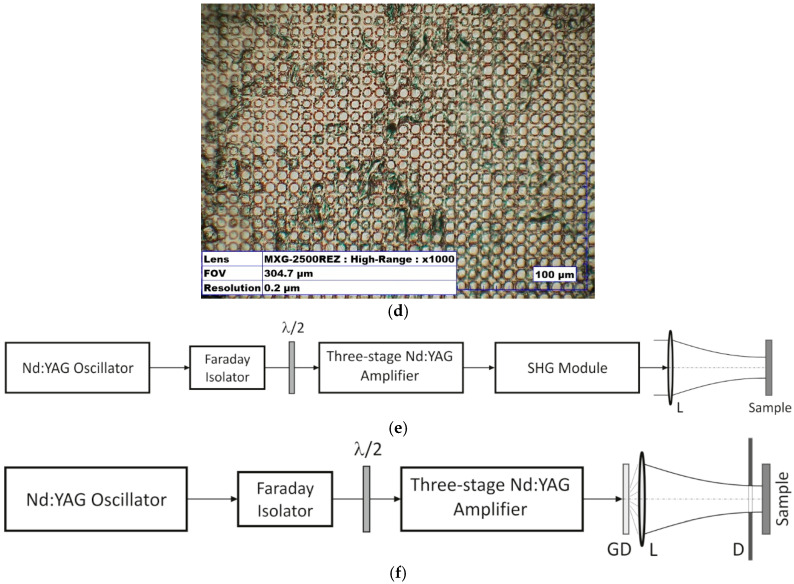
(**a**) Experimental system with prism-based interferometer for tissue acellularisation: A—polarizing attenuator, λ/2—half-wave plate; (**b**) pyramidal prism with a square base; (**c**) microscopic images of the square area of interference pattern (×35); (**d**) its central part at higher magnification (×1000); (**e**) experimental setup: λ/2—half-wave plate, A—polarizing attenuator, L—focusing lens; and (**f**) Experimental setup: M—totally reflecting mirrors, λ/2—half-wave plate, D—diaphragm, GD—glass diffuser, and L—focusing lens.

The direct laser interference lithography (DLIL) method was applied as an unconventional technique of removing cells from tissues to obtain a pure ECM (extracellular matrix completely removed from cells without destroying its structure) [61]. All DLIL experiments were performed in the experimental setup depicted in Figure 2a. The laser system was composed of Q-switched Nd:YAG oscillator emitting pulses at 1064 nm with duration of about 10 ns, three-stage laser amplifier, and the second harmonic generation (SHG) module. The Faraday isolator protected the oscillator against backscattered laser radiation that could perturb its operation. The half-wave plate rotated the polarization plane of radiation and allowed to obtain optimal polarization state on the entrance of the pyramidal prism.

The energy of the pulses was controlled by means of a polarization attenuator (A) inserted at the output of the SHG module. Thanks to this, it was possible to change the input energy to the prism while maintaining a constant laser beam pattern. The energy of pulses at 1064 nm entering the SHG module was constant and amounted to 240 mJ.

Decellularization processes were carried out using the second harmonic of radiation at 532 nm. For the experiments, all tissues were animal-origin and collected on the basis of permission from the bioethics committee by appropriately trained staff. 

The energy of pulses falling on a right square pyramid prism was changed from 20 mJ to over 240 mJ. The diameter of the laser beam was about 10 mm. Four-beam interference technique was implemented by means of a right square pyramid prism with the prism angle of 5°, shown in Figure 2b. The laser beam falling on the prism divided into four beams, which were then directed and superimposed on the sample surface, where they interfered. As a result, an interference pattern was produced that periodically modified the surface (DLIL technique). The period of the interference pattern Λ was, in this case, determined by the wavelength *λ*, the prism angle *α,* and the refractive index *n* of the material from which the prism was made:

(2) $$\Lambda =\frac{\lambda }{2\sin(\arcsin\left(\mathrm{nsin}\alpha \right)-\alpha }$$

For the wavelength of 532 nm, the refractive index was 1.52 and the prism angle 5°; from the dependence, (1) the minimum period of the interference pattern Λ = 8.3 µm was achieved.

Tissue samples were irradiated with the interference distribution in an area of 3.4 mm × 3.4 mm. Such a distribution was also mapped on a test titanium plate with a polished surface. Microscopic images (Hirox KH-8700, Limonest, France) of surface changes as a result of interaction with the interference field are presented in Figure 2c,d. As a result, tissue samples were irradiated with interference dots pattern with repetitive and round spots with a diameter of about 5 μm located ~9.4 μm apart. The slight deviation of the last value from the theoretical one results mainly from the fact that the relationship (1) does not take into account the diffraction effects and the divergence of the laser beam. The total affected area fitted a square of 3.4 mm by 3.4 mm in sizes. 

The obtained interference distribution was then used to irradiate the tissues. In the preliminary tests, the samples were subjected to 1, 3, or 5 laser shots with energy of 10 mJ, 23 mJ, or 50 mJ, at a constant repetition of 1 Hz. For each irradiation condition, two samples were prepared. The tools used in the experiment and the glass substrate (on which the samples were placed) were rinsed with 70% ethanol solution. The material after laser irradiation was placed in a container with 4% formalin. A reference sample was the tissue not exposed to laser radiation.

At first, samples of animal-origin tissue were exposed to 1, 3, 5, or 10 pulses at a constant pulse energy of 240 mJ. Next tissues were exposed to 1, 2, 5, or 10 laser pulses at a constant pulse energy of 215 mJ. For each of the above experimental conditions, 6 to 9 exposures were made, shifting the sample to cover its entire surface. Samples coding and the exposure parameters (wavelength, pulse energy, repetition rate, number of pulses in single exposure) are presented in the Supplementary Materials, Tables S1 and S2. 

Due to the damage to the extracellular matrix, the energy of the pulses falling on the pyramidal prism was reduced (40 mJ, 20 mJ, and 10 mJ) in the last experiment while their number in a single exposure (500, 1000, and 2000 pulses) was increased. Two samples were prepared for each experimental condition. Tools and glass substrate were rinsed with 70% ethanol. The samples after laser irradiation were placed in a container with 4% formalin. All experimental parameters of further irradiation procedure are given in the Supplementary Materials, Table S3 (Parameters of tissue sample irradiation: 1a-8b and 0a-0b—aorta, 8c—aortic valve, 8d—aortic valve leaflet).

##### Gaussian Beam Application

Based on the DLIL experiments, strong light scattering in the tissues was observed when the tissue samples were exposed to interference pattern. The radiation passing through a tissue of only 0.2 mm thick changed the periodic distribution to a homogeneous one. Therefore, in further experiments, it was decided to irradiate the aortic and aortic valve tissue samples with a beam pattern close to Gaussian.

Similarly to DLIL experiments, the laser system was composed of Q-switched Nd:YAG oscillator emitting pulses at 1064 nm with duration about 10 ns, three-stage laser amplifier, and SHG module. The diagram of the experimental system is presented in Figure 2e. The Faraday isolator protects the oscillator against backscattered laser radiation that could perturb its operation. By adjusting the angle of the half-wave plate, it was possible to rotate the polarization plane of laser radiation and maximize the efficiency of the second harmonic conversion in the SHG module.

The laser radiation from the SHG module output or directly from the output of the amplifying assembly was directed to a polarizing attenuator (A) and then to a focusing lens (L) with a focal length of 300 mm. By adjusting the distance of the sample from the lens, the energy density of the laser radiation incident on the tissue was changed. The irradiation of the aortic and aortic valve tissue samples was carried out using laser radiation at basic wavelength of 1064 nm and its second harmonic at wavelength of 532 nm.

For each of the irradiation conditions, two tissue samples were prepared. The tools and glass substrate (on which the sample was placed) were rinsed with a 70% ethanol solution. After laser irradiation, samples were rinsed in saline and then placed in a container with 4% formalin. 

Initially, the pericardium samples were irradiated at a wavelength of 532 nm. The energy of the pulses was constant and equal to 240 mJ, while the fluency and the number of pulses in the single exposure were changed. The names of individual samples and the conditions under which they were irradiated (pulse energy, laser spot size, fluency, repetition rate, number of pulses in single exposure) are summarized in the Supplementary Materials, Table S4 (Parameters of tissue sample exposure to near Gaussian beam). It should be noted that in the last case, corresponding to the highest fluency of 48 J/cm^2^, the tissue sample completely evaporated.

Due to the observed damage to the extracellular matrix, the energy of pulses was reduced to about 60 mJ and then to about 30 mJ to reduce the fluency of radiation incident on tissue samples, while increasing the number of pulses in a single exposure to several hundred. Samples of aortic valves were irradiated with both 1064 nm and 532 nm. In the first case, the laser spot size was 7 mm, and in the second, 8 mm. The sample names and the irradiation process parameters (wavelength, pulse energy, fluency, repetition rate, number of pulses in single exposure) for the 1064 nm and 532 nm cases are given in the Supplementary Materials, Table S5 (Parameters of tissue sample irradiation at 1064 nm: AA—aortic root, XA—aorta, XZ—aortic valve) and Table S6 (Parameters of tissue sample irradiation at 532 nm: AA—aortic root, XA—aorta, XZ—aortic valve), respectively.

##### Top-Hat Beam

The irradiation of aortic tissue and aortic valve tissue samples with laser radiation was carried out using the Nd:YAG laser system [60]. The diagram of the experimental setup is shown in Figure 2f. The Q-switched Nd:YAG oscillator emits laser pulses at the wavelength of 1064 nm with duration about 10 ns. The laser beam is then directed through a Faraday isolator and a half-wave plate to a two-stage amplifier subsystem. The Faraday isolator protects the oscillator against backscattered laser radiation that could distort its operation. The half-wave plate by rotating the polarization plane of radiation allows obtaining optimal *s*-type polarization on the sample surface. The beam pattern at the output of the laser system is close to the Gaussian pattern.

To irradiate tissue samples, the laser beam with an energy distribution in cross-section close to the Gaussian distribution was used [55]. It turned out that in the irradiated tissue samples there were depressions in the vicinity of the beam center, where the laser fluency was the highest. To avoid this, the beam pattern was homogenized by inserting a glass diffuser and a focusing lens with a focal length of 300 mm into the beam path. The task of the diffuser was to scatter the incident laser radiation strongly and thus homogenize its lateral distribution. The task of the lens was to focus the scattered radiation on the sample surface. Additionally, a circular diaphragm with a diameter of 5 mm was placed just above the sample. The area of laser-tissue interaction was thus clearly defined.

In the previous experiment, tissue samples were irradiated with pulse energies of 32 mJ and 65 mJ (the mean fluencies were 83 mJ/cm^2^ and 165 mJ/cm^2^, respectively) [50]. However, in addition to tissue decellularization, some damage to the extracellular matrix was also observed under these conditions. Therefore, in subsequent exposures, the energy in a single pulse (thus reducing the fluency) was reduced, while significantly increasing the number of pulses in a single exposure. All samples were now irradiated with 15 mJ laser pulses, corresponding to the fluency of 76 mJ/cm^2^ at the sample surface.

As damages to the extracellular matrix were observed, in the next stage of the experiment, the energy of the laser pulses was reduced again, this time to 11 mJ, while the number of pulses was increased in a single exposure from 3000 to 5000, and then to 10,000. The aim of these treatments was to avoid damage to the extracellular matrix while maximizing the effectiveness of tissue decellularization. Taking into account the energy of the laser pulse and the area of the laser spot, the fluency at the surface sample in this case was 56 mJ/cm^2^.

It turned out that there were still occasional damages to the extracellular matrix. Therefore, in the next cycle of irradiation of tissue samples, the energy of the laser pulse was further reduced to 10.5 mJ, which corresponded to the lower fluency of 53.5 mJ/cm^2^. The description of tissue samples and the parameters of the irradiation processes (pulse energy, fluency, repetition rate, number of pulses in single exposure) are summarized in the Supplementary Materials, Table S7 (Parameters of tissue sample irradiation: AA—aortic root, ZA—aortic valve.).

During the preparation of the samples for irradiation, the tools and the glass substrate on which the samples were placed were rinsed with a 70% ethanol solution. After the exposure to laser radiation, the samples were placed in a container with an antibiotic bath solution. During longer exposures, it was noticed that the samples were drying up and, therefore, during the irradiation with a series of 10,000 pulses, the tissue was moistened every 10 min with an antibiotic bath solution.

#### 2.2.4. Proton Irradiation

**Figure 3:**
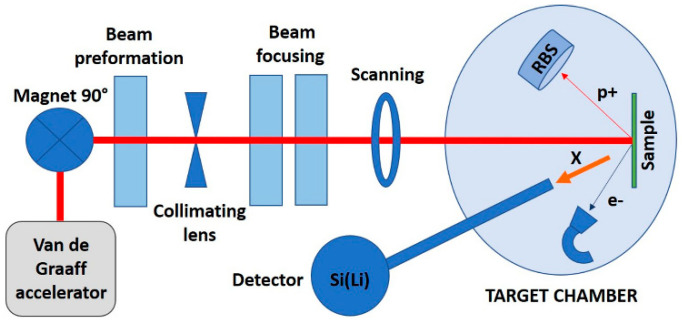
Experimental setup of the Cracow’s scanning nuclear microprobe providing source for proton beam for sample irradiation.

Another unconventional technique applied in attempt to achieve pure decellularized ECM was irradiation by the proton microbeam from the Van de Graaff accelerator. The 2 MeV horizontal focused microbeam facility based on the HVEC K–3000 Van de Graaff accelerator was designed and constructed at the Institute of Nuclear Physics of the Polish Academy of Sciences, Cracow, Poland. Among different radiation types, proton beam deposits most of its energy over a narrow field at the distal end (Bragg peak), therefore, tissues along the entrance and exit path receive relatively low doses. For this reason, proton therapy offers better dose delivery and distribution than conventional photon-based radiotherapy [62]. A simplified scheme of the experimental setup is demonstrated in Figure 3 [63]. During the experiment, samples were irradiated with the 2 MeV protons, with the total beam power of 60 mW distributed over the spot size of ~0.3 mm^2^. The calculated depth of penetration by protons within the irradiated tissue was 62.5 µm, therefore, the energy density was 3 W/mm^3^. Samples were irradiated for five periods of time: 2 s, 5 s, 10 s, 30 s, and 180 s.

### 2.3. Histology and Immunestaining

According to the standard histological techniques, the samples for microscopic examination were fixed for 24 h in 4% buffered paraformaldehyde, dehydrated with increasing concentrations of alcohol, embedded with paraffin, and cut into 6 µm-thick sections using microtome, Wetzlar, Germany. After removing the paraffin, sections were then stained with hematoxylin and eosin (HE) to determine the preservation of general histological structure and to evaluate the efficiency of cell debridement; additionally, the orcein stain was used to assess preservation of the vascular elastic elements. The specimens were examined under an Olympus IX83 light microscope. Images were collected and morphometric analysis was carried out using DP-73 digital CCD camera (Olympus, Tokyo, Japan) and CellSens Dimension (Olympus, Tokyo, Japan) image analysis system.

### 2.4. Principle of Analysis According to an Observer-Blind Mode

The issues raised in this paper are related to aspects of radiobiology. Creating an optimal scaffold for the culture of autologous cells requires development of a new scientific strategies. Materials examined within this study were evaluated in a blind mode, i.e., the researchers did not know how each sample was treated before the tests. However, due to technical limitations, some of the analyses were not fully double-blinded. Tissue samples vary in properties and thickness depending on preparation procedure and where they were taken from. Therefore, only partial control of the experiments was possible. It should be pointed out that this work covers basic research and is aimed at development of an alternative technique, based on physical methods, alternative to the chemical and enzymatic acellularization processes. The object of this research was not only the diagnostics of the irradiated tissues, but most of all it was focused on overcoming the limitations of the currently used techniques in tissue engineering. The work was conducted in two basic stages. In the first stage, laser and proton techniques were developed in terms of their ability to remove cells from tissue samples. This step of the experiments was of a very basic, physical character, focused on the interaction of highly energetic beam and the tissue. The part of work related to the impact of irradiation on the tissue structure was only partly carried out in a blind mode. Changes in tissue structure induced by the beams were accurately recorded. In the second stage, where enzymatic technique was compared with physical techniques, i.e., laser and proton, blind mode was introduced. In Supplementary Materials, the following features were compered and graphically illustrated:

- assessment of optical densities for all techniques that were used to remove cells from tissue;
- percentage of image area not occupied by cells;
- number of cell nuclei per image;
- percentage of imaging area occupied by cell nuclei;
- average size of cell nuclei;
- circularity of cell nuclei.

## 3. Results

### 3.1. FE Modelling of Decellularization Process

**Figure 4:**
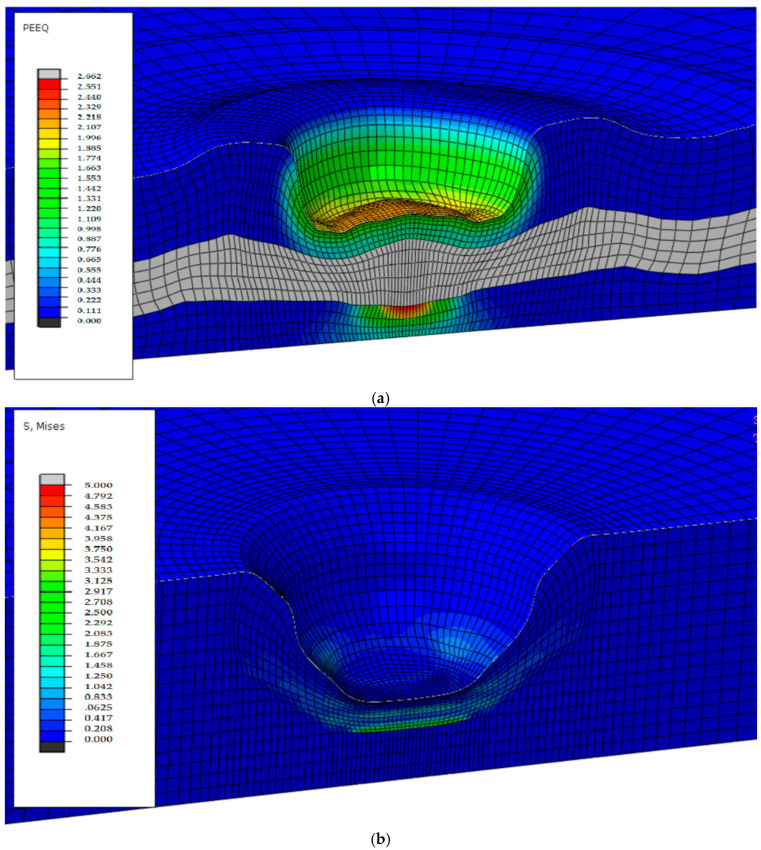

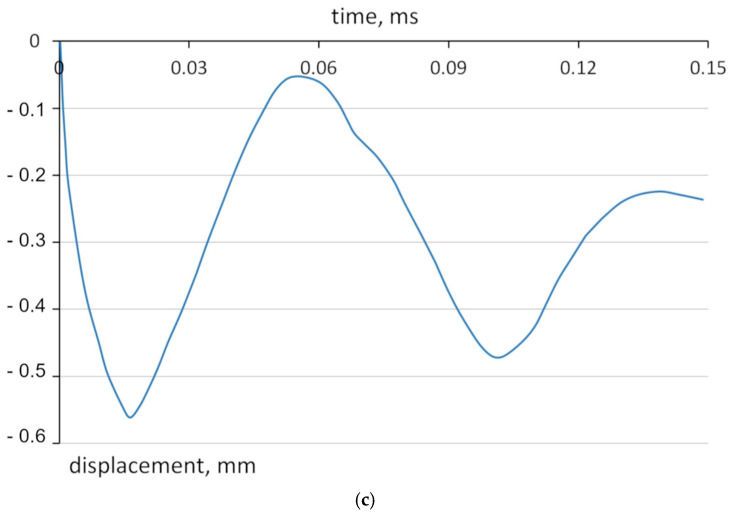
Results of 3D finite element three-layer tissue of animal origin model: (**a**) effective strain distribution—side view in a selected cross-section; (**b**) effective stress distribution—side view in a selected cross-section; (**c**) graph of displacement of central node versus time.

The results of FE model of decellularization process were computed as effective strain and effective stress distributions, as well as a change in the displacement of nodes from the sample center to its edges in time. Figure 4 shows the distribution of the effective strain after the load was applied (Figure 4a), distribution of effective stress as the wave travels into the sample (Figure 4b), and the displacement of the central node in time (Figure 4c). 

The layer of spongiosa in Figure 4a is grayed out because it is not subjected to a plastic deformation. After the wave bounces off the sample as a result of significant deformation of the elements, the effective strain could be 2.5 of that at the bottom of the material layer, as can be seen in Figure 4a, and the mesh is distorted in some places. In the middle of the simulated area, the FE mesh remains practically undistorted, and the effective stress reaches maximum value of 5 MPa. The displacement of the central node in time is of regular type (Figure 4b). The node does not reach displacement above its initial position, while vibrations propagate slowly along the surface of the sample. The maximum value of displacement in the central node is equal to 0.23 mm after loading. The shockwave propagates freely to the edges of the sample, but the FE mesh nodes move especially in the area close to the center of the sample. The average values of displacement measured from the surface to the depth of tissue samples in experimental profilometry tests are also above 0.2 mm. 

Numerical techniques represent the first stage in designing a decellularization process and allow predicting of various physical phenomena occurring at the tissue-laser beam or tissue–proton beam interface. Numerical techniques are particularly useful in establishing the induction of a shock impact on tissue caused by an interference laser beam. This type of phenomenon causes deformation of a piece of tissue at the site of irradiation. 

### 3.2. Tissue Defect Analysis

**Figure 5:**
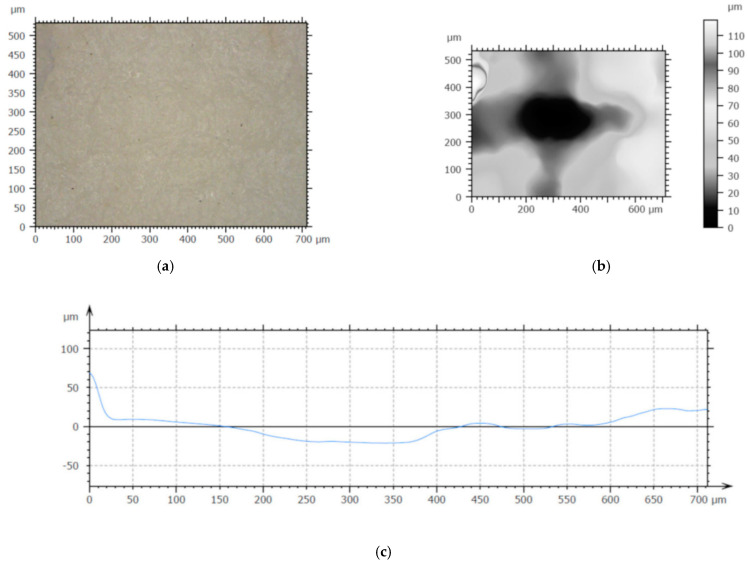
Tissue topography: Z—displacement, mm for animal-origin sample models; (**a**) the surface; (**b**) height map; (**c**) profile [53].

Numerical analysis of mechanical effect of wave causing a decellularization process was verified experimentally. Using the digital microscope, the topography of the tissue was examined in places where it interacted with the laser beam (Figure 5). The strongest decellularization effect was observable at 532 nm wavelength and 32 mJ energy. Tissue tests showed a deflection, in line with predictions, obtained by numerical analysis.

### 3.3. Microscopic Analysis after Proton Irradiation

#### 3.3.1. Laser Influence

**Figure 6:**
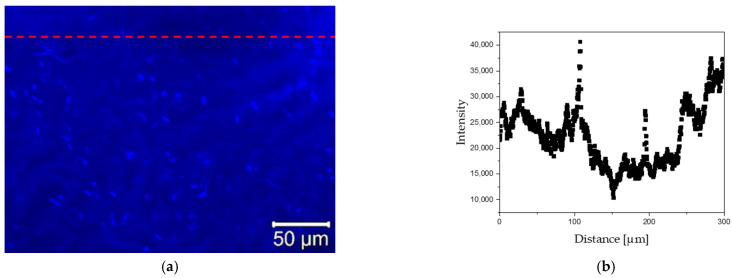
Influence of the laser beam on tissue autofluorescence. (**a**) Autofluorescence and line scan on fluorescence images; (**b**) intensity of light excited in autofluorescence—disappearance of autofluorescence at the laser burning site.

The impact of the laser beam on the tissue is shown in Figure 6. The performed experiments indicated that the application of the laser beam induced shock impact on tissue, which is responsible for the disappearance of autofluorescence signal in tissue during visualization at the site of laser irradiation. This phenomenon is a possible effect of the destruction of tissue elastic fibers.

The disappearance of the autofluorescence signal may be indicative because the elastic elements (elastin proteins) are the main components of vessel walls showing autofluorescence, therefore, the lack of signal may indicate some significant changes in these elements. The histological analysis (see Section 3.3.3) did not show any evident disruption of the elastic elements. Although, it is possible that some molecular changes occurred in the elastin molecules that caused the decrease in autofluorescence signal. Currently, there are no publications directly showing the damaging effect of lasers on the elastic elements. However, some information on the correlation of autofluorescence signal and denaturation process of collagen and elastic fibers can be found in the literature [64,65,66,67,68,69].

#### 3.3.2. Proton Radiation

**Figure 7:**
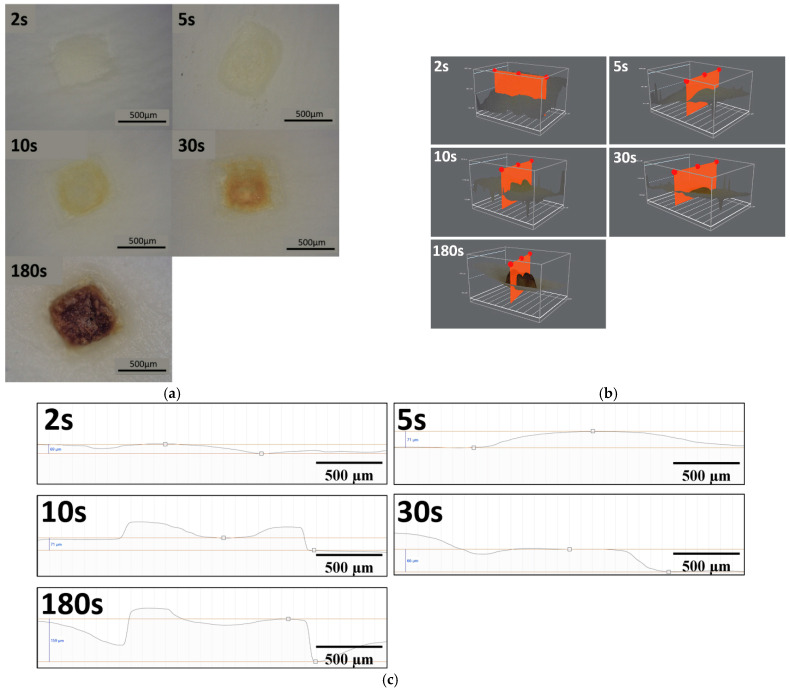
Micrographs of the aortic walls subjected to proton irradiation for exposition time of 2 s, 5 s, 10 s, 30 s, and 180 s; (**a**) top view; (**b**) cross-sectional view; and (**c**) cross-sectional view charts.

Sample irradiation by proton beam was carried out for five periods of time from 2 s to 180 s. Optical observations using digital microscope (Figure 7) indicated significant changes in surface topography for all exposure times. At the point of interaction of the beam and tissue, visible differences between the samples can be noticed in color change, which evolved from pale yellow to dark brown, indicating that the highest dose of radiation (180 s) caused severe damage to the tissue structure (carbonization).

**Figure 8:**
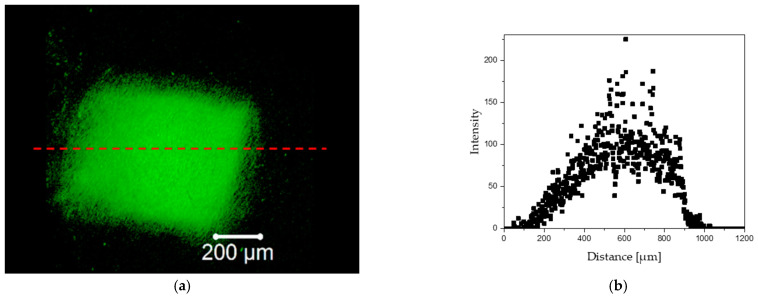
Two second influence of the proton beam on tissue autofluorescence: (**a**) autofluorescence and line scan on fluorescence images; (**b**) intensity of light excited in autofluorescence—increase of autofluorescence at the proton burning site.

**Figure 9:**
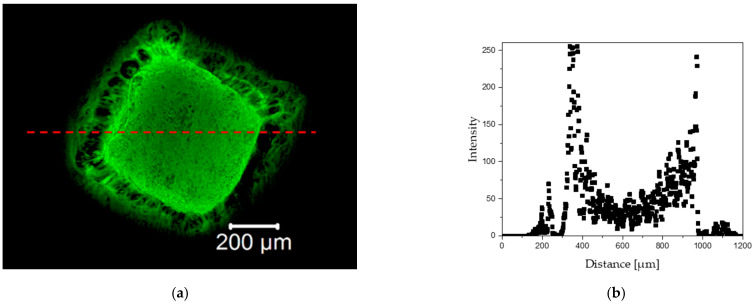
One-hundred-and-eighty second influence of the proton beam on tissue autofluorescence: (**a**) autofluorescence and line scan on fluorescence images; (**b**) intensity of light excited in autofluorescence—increase/decrease of autofluorescence at the proton over burning site.

Figure 8 and Figure 9 show (a) fluorescent images of the tissue sample irradiated with the proton beam and (b) graphical visualization of the light intensity on distance along the red line marked in the fluorescent images. Figure 8 shows the sample irradiated for 2 s and Figure 9 shows the sample exposed to the proton beam for 180 s. Based on the results, loss in autofluorescence signal in the central parts of the irradiated site of the tissue was observed. Hence, it can be argued that longer periods of exposition to proton beam may have a destructive effect on the elastic fibers of the aortic walls.

The phenomenon of increased autofluorescence in tissue samples at the site of irradiation has not been fully understood as yet. It is mostly due to the fact that even very accurate histological techniques do not provide a clear answer. The histological results showed a significant tissue destruction, however, these studies are insufficient to determine unequivocally whether the increase in tissue autofluorescence is due to thickening of the elastic fibers. This is only a hypothesis that will have to be explained experimentally with the support of synchrotron radiation techniques.

#### 3.3.3. Histology

##### Chemical Method

**Figure 10:**
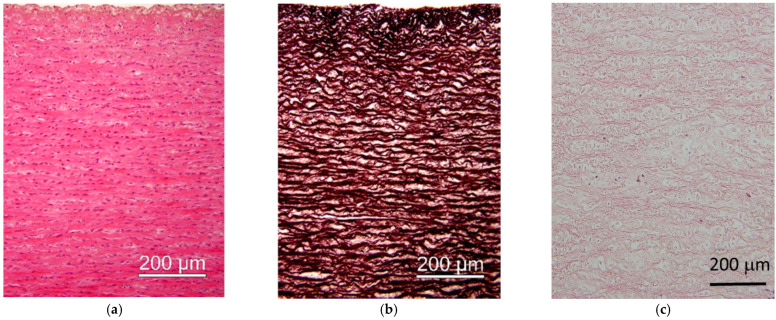
Micrographs of tissue of animal origin (aorta): (**a**) routine (HE) staining showing normal architecture of the media (positive control); (**b**) regular elastic lamellae visualized by staining for elastic element in control sample; (**c**) image of the acellular tissue resulted from the chemical, conventional decellularization technique (HE staining) (negative control).

The decellularization process of the tissue of animal origin with the use of detergent solution was taken as a reference technique to laser and proton beam irradiation. This method effectively removed endothelial and interstitial cells, as well as cell debris in the treated sample. However, the histological analysis showed degradation of the ECM, including swelling of the stroma, disorganization, and/or fragmentation of the fibrous elements (Figure 10c). 

##### Laser

**Figure 11:**
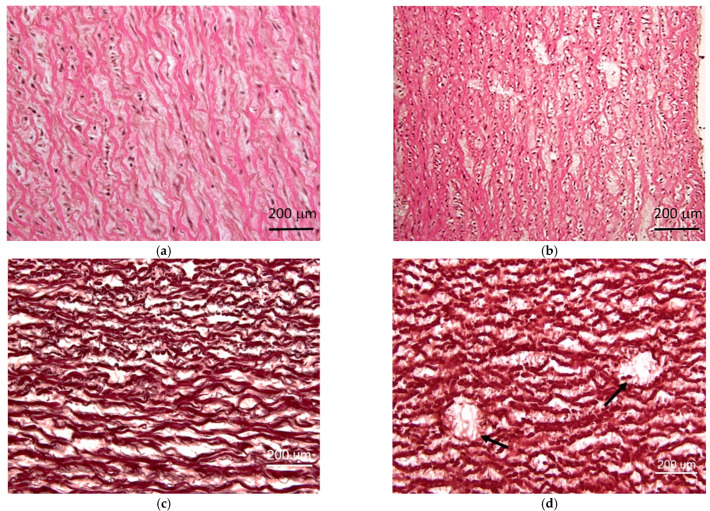
Microscopic images of aortic walls treated with laser beam. The images show: (**a**) mild and (**b**) severe degree of stromal damage with partial cell loss and disorganization (HE staining); (**c**,**d**) specimens stained for elastic elements showing mild (**c**) and severe (**d**) fragmentation of elastic lamellae and focal tissue disintegrations (marked with arrows in (**d**)).

On the contrary to the samples exposed to the chemical decellularization process (Figure 10), only partial elimination of cells and signs of damage of the remaining cells were observed in the case of the laser beam-treated samples (Figure 11). It was manifested by deformation and condensation of the cytoplasm and changes in the pattern of cell nuclei (chromatin condensation/signs of pyknosis). Comparing with the normal (control) tissue, both high and low pulse energy samples showed significantly lower cellularity (20.33 ± 3.12 cells/field vs. 13.17 ± 3.18 vs. 12.14 ± 1.47; mean ± SD for control, high and low pulse energy, respectively; *p* = 0.009, one-way ANOVA test), however, there was no significant difference in respect to the energy used. Connective tissue changes included mainly stromal swelling, disorganization, and/or fragmentation of the fibrous elements. These lesions varied in severity and spread, ranging from local minor damage to significant disorganization involving a large medial area. They were found in 45.2% of the examined samples. In some cases, significant disintegration and weakening of the stroma resulted in the occurrence of focal single or more numerous tears. This form of the lesion (Figure 11d) was observed in 41.9% of examined samples. It was classified as limited, found in a single field of vision (19.4% of cases), or more advanced, found in most of the field of vision (in 22.6% of cases).

In general, the differences between samples were rather small, ranging from virtually no loss of the local cell up to mild degenerative changes in places of the laser beam interaction. There is no evidence that the observed histological differences could be linked to the laser beam settings. 

##### Protons

**Figure 12:**
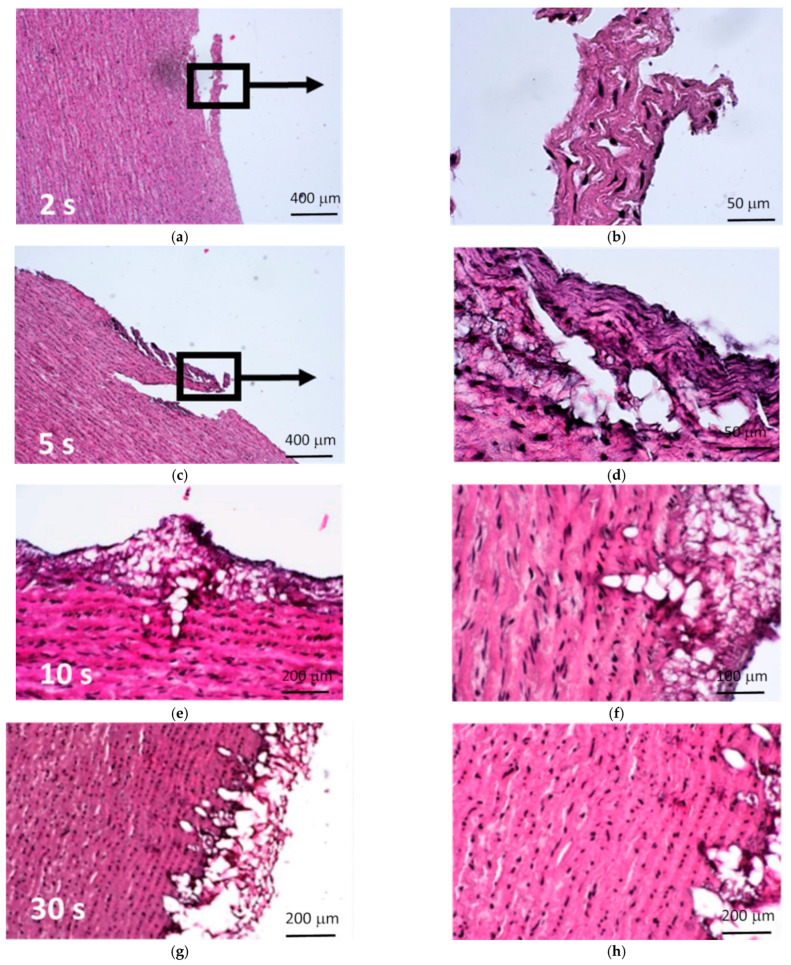

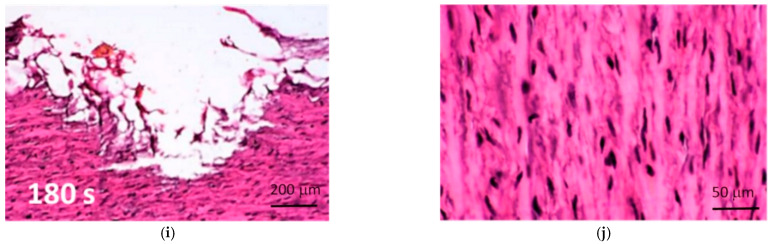
Micrographs of the aortic walls subjected to proton irradiation showing increasing damaging effect of ECM with the time of proton irradiation. Exposition times: (**a**,**b**) 2 s; (**c**,**d**) 5 s; (**e**,**f**) 10 s; (**g**,**h**) 30 s; and (**i**,**j**) 180 s. Right panel shows higher magnifications of the affected areas (HE staining).

Histological images of the samples subjected to beam irradiation are shown in Figure 12. In the tissues exposed to proton beam for 2 and 5 s, the site of irradiation demonstrated little or no effect of decellularization. In the remaining samples (10 s, 30 s, and 180 s), extensive damage to the ECM was observed. Additionally, the extent of it increased with exposure time.

Cell nuclei remained visible in all specimens. At the same time, basophilic (hematoxylin stained) streaks were observed, which were locally present in the samples with shorter exposure times, while in the sample exposed for 180 s they were present over the entire observed area.

For all exposure times, tissues subjected to proton irradiation exhibited changed biological structure. In the area of beam–tissue interaction, severe damage and disintegration of the ECM was observed, which increased with the exposition time. The structure of the ECM for the sample with the longest irradiation time (180 s) was completely disrupted and seemed to be “melted”. In the case of 30 s exposition to proton beam, the connective tissue fibers were deformed and merged. In samples irradiated for 2, 5 and 10 s, a normal pattern of the ECM organization was no longer detectable. 

## 4. Discussion

The decellularization method based on interaction with a proton beam seems to be a promising physical method that can be effectively used to remove cells from a tissue. Proton radiation is normally used for destroying cancerous cells. The biological effect of proton radiation depends on physical factors, such as the geometrical distribution of the radiation dose and biological factors, comprising inter alia, intrinsic radioactivity of cells, oxygenation, position of cells in the life cycle, and degree of hydration [54,55]. Radiation, when penetrating living matter, induces a series of successive processes which vary considerably over time. During interaction of the ionizing radiation beam with living matter, the absorption of radiation energy by atoms and particles along the radiation beam takes place within 10^−18^ s. Absorption of the incident energy of the proton beam causes ionization and excitation phenomena in the targeted tissue. The ionization is more effective as a damaging factor than the excitation because it occurs at energies higher than 10 eV, i.e., corresponding to the protein ionization potential. The ionization probability of the irradiated cells depends on the speed and charge of the ionizing particles and is determined by the Linear Energy Transfer (LPE) coefficient [70]. It refers to the amount of energy lost by the ionizing particle after passing a unit distance in the propagation medium. In order to induce cell death, the radiation energy must be deposited at critical sites in the cells nuclei. There are two possible mechanisms of radiation interaction within the biological shield (being a critical element of the cell): direct and indirect. A direct biological effect refers to an interaction of the radiation with the atoms of the DNA molecule, causing defects that disable cell reproductive potential. The indirect effect is characterized by the fact that the cellular component critical to the cell survival interacts with free radicals. Ionizing radiation causes radiolytic decomposition of water in cells along with the formation of hydrogen and hydroxyl free radicals, which indirectly cause damage to the biological shield. 

In contrast, laser radiation generally does not ionize bio-materials. The energy of the individual quantum is too low to ionize the particles or break the bonds of the molecules. For example, the quantum energy of the fundamental radiation of the most commonly used Nd:YAG laser (at wavelength of 1064 nm) is approximately 1.16 eV and only its fourth harmonic (at 266 nm), in the ultraviolet, have sufficient energy (4.66 eV) to break some chemical bonds [71]. Obviously, it can be easily overcome in the case of very high beam intensities, where non-linear effects begin to dominate. At such situations, multi-photon processes may occur under the interaction of femtosecond pulses, or avalanche ionization may take place with strong focusing of nanosecond pulses. The way in which laser radiation affects biological tissue depends primarily on its intensity, wavelength, and the interaction time. The type of tissue and its optical, thermal, and mechanical properties are also of great importance. It should be noted that laser radiation is scattered from 10 to 100 times more strongly than it is absorbed in biological tissues. Generally, two regimes of laser-tissues interactions are of particular importance: the regime of “low” and the regime of “high” intensity [72]. The absorption and energy dissipation of incident laser radiation results in tissue heating. Even light heating with low intensity pulses can cause cumulative tissue damage due to breaking weak hydrogen or van der Waals bonds, which can lead to the denaturation of proteins and enzymes. Simultaneously, at high intensities of laser radiation (>10^11^ W/cm^2^) optical breakdown, plasma formation and cavitation bubbles become important [73].

Finding a suitable method to obtain a biocompatible scaffold with optimal biomechanical and structural properties is still a challenge in the field of tissue engineering. The literature data [51,52] available so far illustrate the great diversity among the favored methods of tissue removal and crosslinking. This is dictated by different experimental conditions, tissue types, and the purpose for which the scaffold will be used. 

Chemical and enzymatic methods used to remove cells are characterized by high efficiency in cell removal and preservation of the ECM elements. Studies have shown that the biomechanical properties of scaffolds after decellularization differ from those of the native tissue [74]. In this study, an attempt was made to find an alternative to enzymatic and chemical methods. Basing on the obtained results, it is difficult to achieve completely decellularized tissue using proton radiation or DLIL method. On the basis of histological studies, high probability of a destructive effect was found, especially of the laser beam on the elastic fiber structure. However, both methods differ in their impact on the elastic fibers. In the case of the laser method [75], the induced impact on tissue effect is visible, which was manifested by the resulting distortion at the site of beam application and loss of autofluorescence signal. Extinction of the latter effect indicates destruction of the matrix fiber structure. These observations were confirmed by the histological studies.

As a result of the numerical simulations, only the top part of the model of tissue of animal origin is irreversibly damaged, as it is for the tissue sample in the experiment of decellularization process. The maximum values of displacement measured in the experiment are similar to values computed in simulations of decellularization process. The resilient layer of gel spongiosa in combination with two other shock-absorbing layers protects the interior of the valve tissue against damage. The possibility of damping the stresses arising on the sample surface results from the application of the three-layer tissue model of sample and hyperelastic material models, which improve the fluidity and flexibility of the FEM tissue model. This approach is expected to lead to the creation of functional tissue and avoiding the ethical problem associated with removing organs from deceased persons. This solution would also reduce waiting time for a proper vascular or valve implant. Similar computational results were obtained for a simulation of mechanical decellularization process of a single layer tissue sample in [53] applying the laser method. Another simulation of mechanical decellularization process was also presented in [76], however, in that case, sonication-assisted methods were applied.

In the case of the proton method, the irradiated area shows an increase in autofluorescence signal. This phenomenon can be explained by the lack of destructive influence on the fiber structure, while the proton beam can influence the thickening of the structure at the irradiation site. This was confirmed by tissue structure analysis. 

Both methods showed some destructive effect on the genetic material of the nuclei, which was visible as blurred areas within the cell nucleus. Simultaneously, it did not show a sufficient decellularization effect. Even so, it does not eliminate these techniques from tissue removal processes. Both laser and proton physical techniques require further refinement and optimization in the context of tissue interaction and for the time being may represent a hybrid option to the chemical technique.

## 5. Conclusions

This work considers a new physical approach to decellularization processes using laser and proton radiation. The applied physical methods to obtain a decellularized tissue scaffold differed in the efficiency of removing cells from the tissue in favor of the laser irradiation. However, both laser and the proton method had a destructive effect on various extracellular tissue components. The physical techniques investigated within this study require further optimization in the context of interaction with tissue, yet, for the time being may represent a hybrid option to the chemical technique. The FEM 3D simulation of decellularization process of the three-layer origin tissue sample reflects well the mechanical response under a shock impact on tissue loading described by hyperelastic material models and gives results comparable to the experimental ones. The farsighted social aspect of this work is to develop the appropriate substrate dedicated for organ transplantation, basing on a bioactive tissue-based scaffold populated by cells of a graft recipient.

## 6. Patents

From the work reported in this manuscript there is a patent, patent no. P.426790 which was granted in 2021. 

## Figures and Tables

**Table 1:** Hyperelastic and elastic parameters for the tissue of animal origin materials.

Tissue	Young’s Modulus (MPa)	Hyperelastic Properties	
Fibrosa	1.841	Ogden model, N = 1	
		*μ*_1_ = 0.008	*α*_1_ = 25
Spongiosa	0.809	Ogden model, N = 3	
		*μ*_1_ = −0.326	*α*_1_ = 14.675
		*μ*_2_ = 0.298	*α*_2_ = 14.796
		*μ*_3_ = 0.043	*α*_3_ = 11.981
Ventricularis	1.622	Ogden model, N = 3	
		*μ*_1_ = −18.610	*α*_1_ = 18.231
		*μ*_2_ = 10.025	*α*_2_ = 18.778
		*μ*_3_ = 8.606	*α*_3_ = 17.532

## Data Availability

There was no need to use additional databases.

## Supplementary Materials

The following supporting information can be downloaded at: https://www.mdpi.com/article/10.3390/ma15072594/s1, Table S1: Parameters of pericardium sample irradiation; Table S2: Parameters of pericardium and aortic valve sample irradiation; Table S3: Parameters of tissue sample irradiation: 1a-8b and 0a-0b—aorta, 8c—aortic valve, 8d—aortic valve leaflet; Table S4: Parameters of pericardium sample exposure to near Gaussian beam; Table S5: Parameters of tissue sample irradiation at 1064 nm: AA—aortic aorta, XA—aorta, XZ—aortic valve of the aorta; Table S6: Parameters of tissue sample irradiation at 532 nm: AA—aortic root XA—aorta, XZ—aortic valve; Table S7: Parameters of tissue sample irradiation: AA—aorta, ZA—aortic valve. The Supplementary Materials present the following features: (1) assessment of optical densities for all techniques that were used to remove cells from tissue; (2) percentage of image area not occupied by cells; (3) number of cell nuclei per image; (4) percentage of imaging area occupied by cell nuclei; (5) average size of cell nuclei; (6) circularity of cell nuclei.
