# Supplementary material for: Interdisciplinary Methods for Zoonotic Tissue Acellularization for Natural Heart Valve Substitute of Biomimetic Materials
Source: Materials (Basel). 2022 Apr 1;15(7):2594. doi: 10.3390/ma15072594 (PMC9000896; doi:10.3390/ma15072594)
Supplement: Supplementary file 1 [file materials-15-02594-s001.zip › materials-1628293-supplementary.pdf]

materials

MDPI

Supplementary Materials

Table S1. Parameters of pericardium sample irradiation.

Sample nr	Wavelength (nm)	Pulse Energy (mJ)	Repetition rate (Hz)	Number of pulses in single exposure
1				
2			1	1
3	532	215		
4				
5				
6	532	215	1	2
7				
8				
9				
10	532	215	1	5
11				
12				
13	532	215	1	10

Table S2. Parameters of pericardium and aortic valve sample irradiation.

Sample nr	Wavelength (nm)	Pulse Energy (mJ)	Repetition rate (Hz)	Number of pulses in single exposure
1				1
2	532	240	1	3
3				5
4				10
5				1
6	532	240	1	3
7				5
8				10
9				1
10	532	240	1	3
11				5
12				10
13				1
14	532	240	1	3
15				5
16				10

Table S3. Parameters of tissue sample irradiation: 1a-8b and 0a-0b – aorta, 8c – aortic valve, 8d – aortic valve leaflet.

Sample nr	Wavelength (nm)	Pulse Energy (mJ)	Repetition rate (Hz)	Number of pulses in single exposure
1a<br>1b<br>2a<br>2b	532	40	5	500<br>1000
3a<br>3b<br>4a<br>4b<br>5a<br>5b<br>6a<br>6b<br>7a<br>7b<br>8a<br>8b<br>8c<br>8d	532	20<br>10	5	500<br>1000<br>2000<br>500<br>1000<br>2000
0a<br>0b	Native tissue (without irradiation)			

Table S4. Parameters of pericardium sample exposure to near Gaussian beam.

Sample nr	Pulse Energy (mJ)	Laser spot size (mm)	Fluency (J/cm²)	Repetition rate (Hz)	Number of pulses in single exposure
1		4.8	1.3		4
2		2.3	5.7		4
3	240	2.3	5.7	1	2
4		2.3	5.7		2
5		0.8	48		2

Table S5. Parameters of tissue sample irradiation at 1064 nm: AA - aortic aorta, XA - aorta, XZ - aortic valve of the aorta.

Sample nr	Wavelength (nm)	Pulse Energy (mJ)	Fluency (J/cm²)	Repetition rate (Hz)	Number of pulses in single exposure
AA1					100
AA2					50
XA3				5	10
XA4	1064	65	0.17		200
XA5					500
XA6				1	100
XZ7				5	100
XZ8	1064	32	0.08	5	100
AA9					100

AA10				1	100
AA11					200
AA12				5	200
AA13					500
AA14					50
AA15					10

Table S6. Parameters of tissue sample irradiation at 532 nm: AA - aortic root XA - aorta, XZ - aortic valve.

Sample nr	Wavelength (nm)	Pulse Energy (mJ)	Fluency (J/cm²)	Repetition rate (Hz)	Number of pulses in single exposure
AA16	532	62	0.12	5	500
AA17					200
AA18					100
AA19				1	100
AA20				5	50
AA21					10
XZ22					100
AX23					500
AX24					200
AX25					100
AX26				1	100
AX27				5	50
AX28					10
AX29	532	32	0.06	5	500
AX30					200
AX31					100
AX32				1	100
AX33				5	50
AX34					10
AX35					500
AA36					200
AA37					100
AA38				1	100
AA39				5	50
AA40					10

Table S7. Parameters of tissue sample irradiation: AA – aorta, ZA - aortic valve.

Sample nr -1-	Pulse Energy (mJ) -2-	Fluency (J/cm²) -3-	Repetition rate (Hz) -4-	Number of pulses in single exposure -5-
AA0	Native tissue			
AA1	15	76	1	600 1000
AA2				
ZA3				

ZA4				
ZA5	Reference sample – native valve			
AA6	Reference sample – native tissue			
AA7<br>ZA8<br>ZA9<br>ZA10	15	76	2	2000
AA11<br>ZA12	15	76	5	2000
AA13<br>ZA14	15	76	5	3000
AA15<br>ZA16	11	56	5	3000
AA17<br>AA18<br>AA19<br>AA20<br>AA21<br>AA22<br>AA23<br>AA24<br>AA25<br>AA26<br>AA27<br>AA28<br>ZA29	11	56	5	3000
AA30<br>AA31<br>AA32<br>AA33<br>AA34<br>AA35<br>ZA36	11	56	5	10000
AA37<br>AA38<br>AA39<br>AA40<br>AA41<br>AA42	10.5	53.5	5	3000
AA43	10.5	53.5	5	5000
